# Possible implication of oxidative stress in anti filarial effect of certain traditionally used medicinal plants *in vitro* against *Brugia malayi* microfilariae

**DOI:** 10.4103/0974-8490.75453

**Published:** 2010

**Authors:** R. D. Sharma, A. R. Veerpathran, G. Dakshinamoorthy, K. N. Sahare, K. Goswami, M.V.R. Reddy

**Affiliations:** *Department of Biochemistry & JB Tropical Disease Research Centre, Mahatma Gandhi Institute of Medical Sciences, Sevagram, Wardha, India*

**Keywords:** Medicinal plants, oxidative stress, antifilarial effect, *B. malayi*

## Abstract

**Introduction:**

Tropical disease research scheme of World Health Organization has duly recognized traditional medicine as alternative for antifilarial drug development. Polyphenolic compounds present in traditionally used herbal medicines are natural antioxidants; however, paradoxically they may exert pro-oxidant effect. Popular drug diethyl carbamazine citrate harnesses the innate inflammatory response and the consequent oxidative assault to combat invading microbes.

**Methods:**

With this perspective, extracts of *Vitex negundo* L. (roots), *Butea monosperma L.* (leaves), *Aegle marmelos Corr.* (leaves), and *Ricinus communis* L. (leaves) were selected to explore the possible role of oxidative rationale in the antifilarial effect *in vitro*.

**Results:**

Apart from the last, other three plant extracts were reported to have polyphenolic compounds. Dose-dependent increase was found in the levels of lipid peroxidation and protein carbonylation for all the three plant extracts except *Ricinus communis* L. (leaves). Such increase in oxidative parameters also showed some degree of plant-specific predilection in terms of relatively higher level of particular oxidative parameter. High degree of correlation was observed between the antifilarial effect and the levels of corresponding oxidative stress parameters for these three plants. However, extracts of *Ricinus communis* L. (leaves) which is relatively deficient in polyphenolic ingredients recorded maximum 30% loss of motility and also did not show any significant difference in various stress parameters from corresponding control levels.

**Conclusion:**

These results reveal that targeted oxidative stress might be crucial in the pharmacodynamics.

## INTRODUCTION

In the tropical region, microbial diseases are of considerable concern. The International Task Force for Disease Eradication has identified filariasis as one of the six major infectious diseases and consequently demands serious thrust in development of novel drugs. Since the last century, Diethyl carbamazine citrate (DEC) has been almost the sole antifilarial drug. The mechanism of action of DEC is still not very clear. It is supposed to induce macrophages for releasing various mediators belonging to reactive oxygen species.[1] This provides a clue toward development of novel antifilarial therapeutic rationale.

Tropical disease research scheme of World Health Organization recognized traditional medicine as important alternative source for novel antifilarial therapeutics.[2] However, the major challenge in the use of traditional medicines is the lack of scientific validity. Hence, we took interest in certain traditionally described antifilarial herbs for exploring their possible mechanism of action. Earlier work with extracts of *Vitex negundo* L. (Nirgundi roots), *Butea monosperma L.* (Palas leaves), and *Aegle marmelos Corr*. (Beal leaves) validated their antifilarial efficacy *in vitro*; another herbal extract, *Ricinus communis L*. (Erandi leaves), with known antifilarial effect was also tested, which however showed meager response.[3] Later work confirmed that herbal extracts of *Vitex negundo* L. (roots) and *Aegle marmelos Corr*. (leaves) contain important polyphenolic compounds like flavonoids or coumarins,[4] whereas *Butea monosperma L.* (leaves) was found to have saponins. On the contrary, Ricinus plant did not contain any major polyphenolics. Recent evidences showed that polyphenolic compounds in plants like flavonoids, in a role reversal format, might behave as pro-oxidants rather than antioxidants.[5]

With this perspective, the present study was conducted with the above mentioned extracts to look out for the possible involvement of any oxidative mechanism in the antifilarial action of herbal extracts which were traditionally used as antifilarial therapeutics.

## MATERIALS AND METHODS

### Plant extracts acquisition

On the basis of earlier work with *Vitex negundo* L. (Nirgundi roots, voucher no. 9022), *Butea monosperma* L. (Palas leaves, voucher no. 9024), and *Aegle marmelos* Corr. (Bael leaves, voucher no. 9023), extracts were selected for the study.[3] *Ricinus communis* L. (Erandi leaves) that showed relatively weaker efficacy was also included for comparative assessment. These plants were procured from the local areas and were confirmed by a taxonomist (Voucher specimens stored at Dept. of Botany, RTM Nagpur University, Nagpur).

### Preparation of herbal extracts

The herbal extracts of the referred plants were prepared following standard protocol as described earlier.[3] These compounds were further used for the phytochemical analysis, which revealed the presence of alkaloids, saponins, and flavonoids in *Vitex negundo* root extracts, and coumarins in *Aegle marmelos* leaf extracts.[4] Similarly, saponins were found to be present in *Butea monosperma* leaf extracts.[6]

### Preparation and collection of *B. malayi* microfilariae (Mf)

Microfilariae (Mf) of B. *malayi* were obtained by lavage from the peritoneal cavities of infected jirds (*Meriones unguiculatus*). The Mf were collected, washed with RPMI 1640 medium (with various supplements such as gentamicin, 20 µg; penicillin, 100 µg; streptomycin, 100 µg; 15 mM Hepes, NaHCO_3_, 29 mg; organic acids [malic acid, α ketoglutaric acid, D-succinic acid, and fumaric acid at concentrations of 670, 370, 60, and 55 mg/l, respectively] and sugars [sucrose and fructose at concentrations of 26,680, and 400 mg/l, respectively] of Graces insect culture medium in 1 l of sterile triple distilled water and sterilized by filtration through 0.22 µm membrane) and used for *in vitro* experiments. The use of animals for the study was approved by Institutional Animal Ethical Committee which follows CPCSEA norms.

### *In vitro* screening of herbal extracts for antifilarial activity

Crude extracts of all plants except *Ricinus communis* L. (leaves) were diluted in suitable solvents[3] to obtain the desired final concentration range of 20 to 100 ng/ml in sterile 24 well culture plates (Nunc, Denmark) containing 900 µl of RPMI media. Extract of *Ricinus communis* L. (leaves) which showed relatively less significant therapeutic efficacy in earlier screening[4] was used only in the highest concentration (100 ng/ml). Wells without any extract but with similar solvents in 900 µl of the media were kept as corresponding controls. Approximately, 100 Mf in 100 µl of RPMI media were introduced into each well for every test sample and also for corresponding control samples (each individual samples in triplicates). The plates were incubated at 37°C for 48 hours in CO_2_ (5%) incubator. Mf motility was assessed by microscopy after incubation; the observations were recorded as the number of nonmotile Mf of the total 100 parasites in each well as percentage. These conditions of assay procedure were standardized in our lab.[3] Each experiment (in triplicate aliquots) was repeated thrice to check the reproducibility. We had restricted the experiments only on the Mf for testing the efficacy of the drugs, considering the technical difficulties in procuring adult worms.

Culture supernatants from each of the herbal extracts obtained after centrifugation (2500 rpm for 10 min) were used for measurement of various oxidative stress markers like lipid peroxidation (MDA) and protein carbonylation.

### Estimation of malondialdehyde (MDA)

Lipid peroxidation was measured using thiobarbituric acid (TBA) method.[7] To 0.5 ml of culture supernatant, 2.5 ml of 10% Trichloroacetic acid (TCA) and 1 ml of 0.67% TBA were added, mixed and boiled in water bath for 30 min. After cooling, resultant chromogen was extracted in 4 ml n-butyl alcohol by separation of organic phase with centrifugation at 3000 rpm for 10 min. Absorbance was measured at 530 nm and values compared against the control n-butyl alcohol. The concentrations of MDA in samples were calculated using standard curve.

### Assay for carbonylation

For protein carbonylation estimation,[8] the samples treated with 10% TCA were made to react with 0.5 ml of DNPH (in 2M HCl) at room temperature for 1 hour. The pellet yielded after precipitation with ice cold 10% TCA and centrifugation at 5000 rpm for 5 min was washed thrice with ethanol-ethyl acetate (1 : 1). The washed pellet was dissolved in 1.5 ml of protein-dissolving solution (2 g SDS and 50 mg EDTA in 100 ml 80 mM phosphate buffer of pH 8) and kept at 37°C for 10 min. The color intensity was measured at 370 nm against 2 M HCl. Carbonyl content was calculated by using molar extinction–coefficient (21 × 10^3^ l/mol/cm).

### Statistical analysis

Pearson’s correlation analysis between each of the oxidative parameters and the corresponding dose-dependent loss of microfilarial motility was carried out for three herbal extracts individually.

## RESULTS

The result showed dose-dependent loss of microfilarial motility used for the three herbal extracts namely *Vitex negundo* L. roots, *Butea monosperma* L. leaves, and *Aegle marmelos* Corr. leaves. All these values recorded were remarkably higher over the entire dose range as opposed to the corresponding control levels [Table 1]. Also, correspondingly high ascending trend in the levels of MDA and carbonyl content of protein in culture supernatant preincubated with these three herbal extracts were recorded. The observed change in the levels of these parameters showed plant specificity [Figure 1a and b]. *Vitex negundo* L. roots showed very steep dose-dependent increase in carbonyl content of protein, which was lower for MDA level, whereas *Butea monosperma* L. leaves showed only a dose-dependent increase in MDA levels but not in carbonyl content. High correlation coefficients were found between such increase in the various oxidative parameters and the corresponding loss of microfilarial motility [Table 2].

**Table 1:** Effect of the crude herbal extracts on *B. malayi* microfilariae at different concentrations after incubation for 48 hr

Concentration (ng/mL)	*Vitex negundo* roots	*Butea monosperma* Leaves	*Aegles marmelos* leaves
20	10.11 ± 0.11(1.65)	14.41 ±0.15(3.1)	11.23 ± 0.21(1.8)
40	15.3 ±0.16(2.5)	17.68 ± 0. 18(3.8)	16.15 ± 0.11(2.5)
60	25.00 ± 0.25(4.08)	27.33 ± 0.29(5.9)	28.00 ± 0.26(4.4)
80	47.21 ± 0.49(7.7)	42.18 ± 0.43(9.1)	75.67 ± 0.67(11.9)
100	98.96 ± 0.93(16.2)	99.98 ± 0.78(21.5)	97.99 ± 0.86(15.4)
Control 1	6.12 ± 0.06	-	-
Control 2	-	4.65 ± 0.46	-
Control 3	-	-	6.36 ± 0.63

**Table 2:** Pearson Correlation analysis between various oxidative stress parameters and loss of motility induced by various plants extracts upon B. malayi microfilariae.

Plant extracts tested	MDA Levels	Carbonyl content
*Butea monosperma* L. (leaves)	0.756	0.852
*Vitex negundo* L. roots	0.628	0.898
*Aegle marmelos Corr*. leaves	0.646	0.782

**Figure 1(a):**
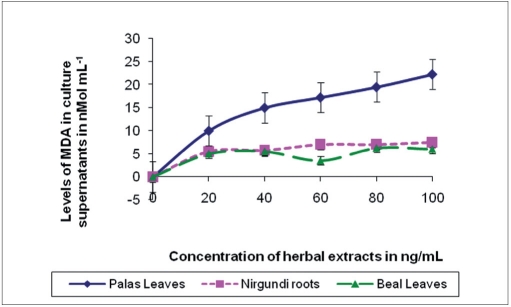
Fold increase in levels of MDA in the culture supernatants after 48 hr incubation in the *in vitro* experiments (relative to controls) for the effect of the herbal extracts on micro filarial motility. Results are from three individual experiments and the levels were expressed in nM mL-1.

**Figure 1(b):**
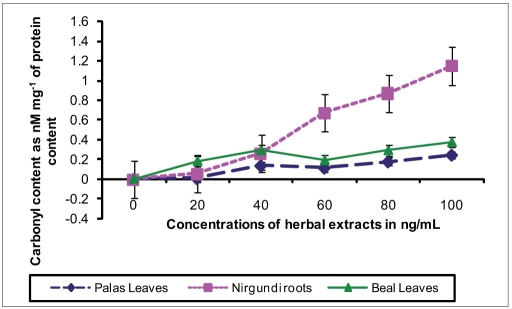
Fold increase in levels of carbonyl content in the culture supernatants after 48 hr incubation in the *in vitro* experiments (relative to controls) for the effect of the herbal extracts on micro filarial motility. Results are from three individual experiments and the levels were expressed in nM mg-1 of total protein content.

Extract of *Ricinus communis* L. leaves showed merely 30% loss of microfilarial motility against 100% loss of motility in the other three herbal extracts used in similar dose (100 ng/ml). The observed differences in the oxidative stress parameters with *Ricinus communis* L. (leaves) extract were not found to be considerably different (6 ± 0.72 ng/mlfor MDA and 6.63 ± 0.83 nM/mg of protein for carbonyl content, respectively) from the corresponding control levels (5.75 ± 0.58 ng/mlfor MDA and 6.45 ± 0.71 nM/mg of protein for carbonyl content, respectively).

## DISCUSSION

Certain plants used in traditional medicine tested in this study namely *Vitex negundo* L. roots, *Butea monosperma* L. leaves, and *Aegle marmelos* leaves were reported to contain various polyphenolic compounds as active ingredients. *Vitex negundo* belonging to Verbenaceae family contain active ingredient vitexicarpin, a flavone analogue.[9] Similarly, *Butea monosperma* from Fabaceae family is also known to contain flavonoids like butrin and isobutrin as active ingredients.[10] As a representative of Rutaceae family, *Aegle marmelos* contain rutin, a flavonoid derivative.[11] It also contains coumarins that come under the broad group of polyphenolic compounds.[13] Previous work on preliminary phytochemical analysis has also supported the presence of similar polyphenolic derivatives in *Vitex negundo* and *Aegle marmelos* extracts namely flavonoids and coumarins, respectively.[4] On the contrary, *Ricinus communis*, a member of Euphorbiaceae group of plants reported to contain potential toxin (ricin), a lectin capable of hemagglutination and a long chain fatty acid (Ricinoleic acid).[14] Though there is report that some bioflavonoids could be recovered from Ricinus leaves, which showed to have insecticidal property,[15] the main component, ricin is not related to phenolic compounds. The antifilarial effect found in this study showed dose-dependent significant increase in the loss of motility of the parasite for all three plant extracts but not with the latter plant (*Ricinus communis*); similarly, statistically significant correlation between oxidative stress parameters with corresponding antifilarial effect was recorded only for the first three plant extracts but not with the latter. Moreover, we did not record any major difference in the levels of oxidative stress parameters from the respective control levels in case of *Ricinus communis* L. (leaves) extract. As discussed above, Ricinus is relatively less resourceful in terms of bioflavonoids/polyphenolic compounds as compared with the other plants used in this study with active polyphenolic constituents. This corroborates well with the observed subdued antifilarial effect of Ricinus and also hints towards the causal association of polyphenolics in such effect. Although polyphenolics are conventional antioxidants,[16] due to the reversible nature of redox reactions and documented dual behavior of myriad polyphenolic compounds in terms of redox activity,[5] an underlying oxidative nexus can be envisaged in the pharmacodynamics.

Later work showed the presence of saponins in *Butea monosperma* leaf extracts (unpublished observation), which is also supported by other studies.[6] In the present study, we observed very high level of lipid peroxidation which further increased in dose-dependent manner for the samples treated with extracts of *Butea monosperma*, whereas such up-surge in carbonylation of protein was found with *Vitex negundo* L. roots. Presence of saponins in *Butea monosperma* leaf extracts might explain such augmented lipid peroxidation, as saponins have been referred to enhance membrane lipid peroxidation.[17] On the other hand, polyphenolic agents like flavonoids in *Vitex negundo* roots may be responsible for such higher level of protein oxidation.[18] Significant correlation between each of these parameters and the loss of microfilarial motility over the dose range indicate an underlying influence of such oxidative macromolecular damage in the antifilarial effect of these plants.

*In vivo* myriad proinflammatory molecules released against invading pathogen to induce inflammatory response act mainly as oxidative stress mediators. Hence, our result suggests that these herbal agents might be imparting the microbial damage through similar mechanism in an innate immune response-mimetic manner. Quite strikingly, popular antifilarial agent, DEC fails to show any *in vitro* effect; however, *in vivo* environment appears to be conducive for its action because of the involvement of immunocytes[19] which subsequently may develop supposedly similar proinflammatory oxidative milieu. Hence, it can be surmised that the final antimicrobial effect might exploit the oxidative route. However, filarial parasite is otherwise reported to be resistant toward oxidative stress.[20] In this context, it is worth mentioning that in our earlier *in vitro* study, H_2_O_2_ (a potent oxidant) though appeared to be less effective alone at lower dose, however managed to bring forth remarkable antifilarial effect with DEC which is also devoid of any individual *in vitro* effect.[21] It is interesting to note that of late, DEC has been shown to have apoptotic effect on filarial parasite *in vitro*.[22] Because there is established relationship between oxidative stress and apoptosis,[23] it is quite tempting to speculate such *modus operandi* in the observed pharmacological response associated with oxidative stress in this study. Pending direct evidential support for such effect, this study unravels a novel mode of antifilarial drug designing based on targeted oxidative rationale.
